# Strategies to identify and edit improvements in synthetic genome segments episomally

**DOI:** 10.1093/nar/gkad692

**Published:** 2023-08-24

**Authors:** Alexandra Rudolph, Akos Nyerges, Anush Chiappino-Pepe, Matthieu Landon, Maximilien Baas-Thomas, George Church

**Affiliations:** Department of Genetics, Harvard Medical School, Boston, MA 02115, USA; Department of Genetics, Harvard Medical School, Boston, MA 02115, USA; Department of Genetics, Harvard Medical School, Boston, MA 02115, USA; Wyss Institute for Biologically Inspired Engineering, Boston, MA 02115, USA; Department of Genetics, Harvard Medical School, Boston, MA 02115, USA; Department of Genetics, Harvard Medical School, Boston, MA 02115, USA; Department of Genetics, Harvard Medical School, Boston, MA 02115, USA; Wyss Institute for Biologically Inspired Engineering, Boston, MA 02115, USA

## Abstract

Genome engineering projects often utilize bacterial artificial chromosomes (BACs) to carry multi-kilobase DNA segments at low copy number. However, all stages of whole-genome engineering have the potential to impose mutations on the synthetic genome that can reduce or eliminate the fitness of the final strain. Here, we describe improvements to a multiplex automated genome engineering (MAGE) protocol to improve recombineering frequency and multiplexability. This protocol was applied to recoding an *Escherichia coli* strain to replace seven codons with synonymous alternatives genome wide. Ten 44 402–47 179 bp *de novo* synthesized DNA segments contained in a BAC from the recoded strain were unable to complement deletion of the corresponding 33–61 wild-type genes using a single antibiotic resistance marker. Next-generation sequencing (NGS) was used to identify 1–7 non-recoding mutations in essential genes per segment, and MAGE in turn proved a useful strategy to repair these mutations on the recoded segment contained in the BAC when both the recoded and wild-type copies of the mutated genes had to exist by necessity during the repair process. Finally, two web-based tools were used to predict the impact of a subset of non-recoding missense mutations on strain fitness using protein structure and function calls.

## Graphical Abstract

**Graphical Abstract:**
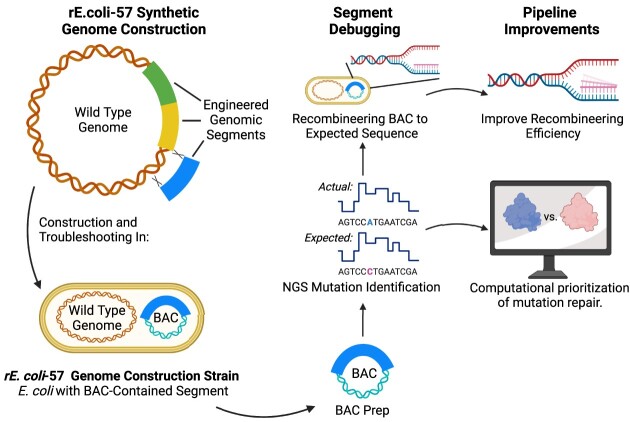

## INTRODUCTION

Whole-genome engineering provides scientists with unique opportunities to explore and expand the possibilities afforded to biological organisms. While the limit to number, extent and purpose of changes that can be made to the genome when engaging in such projects is—theoretically and excitingly—limitless, this work occurs in the context of developing a recoded *Escherichia coli* strain with a seven codon compression scheme, *rE.coli*-57 (1–11). In genome recoding, all instances of one or more codons are replaced with synonymous alternatives and their corresponding translation machinery is removed to prevent recognition of the target codon (1–3). Successfully recoded organisms are resistant to bacteriophage infection and horizontal gene transfer if the foreign DNA introduced contains the removed codons, and recoded organisms allow for strategic reintroduction of the removed codons with corresponding translation machinery encoding non-standard amino acids for biocontainment and protein engineering purposes (1,12–18).

When constructing the first recoded bacterial strain, C321dA, the 321 TAG stop codons in the *E. coli* MG1655 genome were recoded using multiplex automated genome engineering (MAGE) in segment clusters and combined into a single strain using conjugative assembly genome engineering (2,3). However, as researchers recode larger genomes or design more ambitious codon compression schemes, fully recoding a genome using MAGE becomes infeasible. The *rE.coli*-57 genome was designed computationally to optimize synonymous replacement of 62 214 instances of the seven codons from the MDS42 *E. coli* genome (1). To allow researchers to troubleshoot the genome in sections, the recoded genome was then divided into 87 segments, ∼40 genes (or 50 kb) long, that could be constructed from 2–4 kb overlapping Genebytes on bacterial artificial chromosomes (BACs) with a mini-F replication origin (pYES1L-URA) (9).

Each intentionally altered nucleotide in the designed synthetic genome represents a hypothesis about what changes in the final strain will be tolerated. Any rewritten genome may require further optimization, either during strain construction or during strain optimization post-construction, to result in a strain with similar fitness to the wild type or optimized towards a different phenotype (19–21). Furthermore, at any stage in the process of designing modified genomes, synthesizing their DNA, combining them together, adding them into the synthetic genome and outgrowth of strains containing these synthetic constructs, unintentional mutations can be introduced into the final organism's genome (19,22). For large or complex mutations, such as deletions or insertions, resynthesis of *de novo* DNA and Cas9-mediated recombineering of the new construct would be the preferred incorporation method. However, as single nucleotide polymorphisms (SNPs) are the most likely error in DNA synthesis and resynthesized DNA is likely to contain other errors due either to random mistakes or to inherently difficult to synthesize loci (such as repeat regions), recombineering using single-stranded DNA (ssDNA) allows researchers to edit as they go towards strain engineering completion.

Since its description in the scientific literature over two decades ago (23), the use of single strand annealing proteins (SSAPs) to integrate short ssDNA oligonucleotides has undergone significant improvements to increase its efficiency as a genetic engineering tool. These include, but are not limited to, developing a protocol to simultaneously edit multiple sites using MAGE (24), construction of broad-host plasmids with transient inactivation of mismatch repair (the pORTMAGE plasmid system) to enable higher efficiency recombineering across a range of host bacteria strains (25) and systematic identification of SSAPs that improve allelic replacement frequency in *E. coli* cells (26). While recombineering with ssDNA oligonucleotides is a quick method to obtain an edited colony with a low incidence of off-target mutations, improvements to recombineering efficiency prove important when the edit being introduced is deleterious, with unedited bacteria outcompeting edited bacteria. Additionally, the target location or desired edit may require synthesis of an oligonucleotide outside of optimal parameters, such as a particularly low folding energy, presence of a hairpin structure or introduction of an insertion, deletion or multiple edits on the same oligonucleotide that result in decreased homology of the oligonucleotide to the target genomic locus (24,27,28). Thus, characterization of ways to improve recombineering efficiency is particularly important to whole-genome engineering work, where researchers have to work with all loci in the genome, regardless of their level of complexity. Finally, certain applications may involve the presence of two or more loci with high homology to the oligonucleotide, such as when a recoded synthetic segment on a BAC must be edited before its wild-type genomic counterpart is able to be deleted (1,4,12).

BAC recombineering is a well-established technique, especially for manipulating DNA used to develop transgenic animal or cell lines (29–32). Researchers have shown the technique's capacity to incorporate double-stranded DNA cassettes and ssDNA oligonucleotides (33–37). However, its utility in integrating ssDNA oligonucleotides for large-scale genome synthesis and engineering work, for which BACs provide a useful vector to hold and test long stretches of synthetic DNA, has not been well established but could be used to generate mutations that would be advantageous to genome engineering work. Such mutations may include changes to genome segments to improve fitness, diversification to identify viable changes in a particular locus and introduction of small genetic engineering target sites (such as PAM sites and restriction enzyme-cut sites). Further, introduction of target mutations using MAGE makes such processes highly multiplexable. Although many protocol parameters may be adjusted to improve recombineering efficiency, we tested the impact of four parameters on recombineering efficiency for a single recombineering cycle targeting the same locus: *E. coli* genomic background, oligonucleotide direction, SSAP selected and cell density during transformation. The testing of these protocol parameters was applied to the repair of non-recoding mutations in *de novo* synthesized DNA segments used in the construction of a 57 codon-recoded *E. coli* strain, *rE.coli*-57 (1).

In this work, we describe testing recombineering efficiency for cell density during transformation, following testing to ensure selection of an optimal *E. coli* genotype, oligonucleotide direction and pORTMAGE plasmid for introduction of a premature stop codon into the *lacZ* gene. Recombineering was applied to the development of the *rE.coli*-57 strain (1). For this work, 10 segments with a total of 22 non-recoding mutations in essential genes were selected as targets for repair on their BAC vectors using recombineering, allowing these segments to move to the strain assembly pipeline (1). Following repair of these non-recoding mutations, protein structure prediction versus protein sequence conservation (as a proxy for protein function) were tested to determine whether these programs could be used for hypothesis generation to address the question of why certain missense mutations improved complementation fitness. To motivate other researchers to enter the whole-genome engineering field, we use versions of computational tools run from user-friendly interfaces.

## MATERIALS AND METHODS

### Bacterial strains and growth conditions

Three *E. coli* strains were used to complete this project, selected because of their importance in the recombineering literature and the *rE.coli*-57 project: MDS42 [Scarab Genomics, full genotype can be found in Pósfai *et al.* (38)], TOP10 [Invitrogen, Cat. No. C404050, Genotype: F*-mcrA Δ(mrr-hsdRMS-mcrBC) Φ80lacZΔM15 ΔlacX74 recA1 araD139 Δ(araleu)7697 galU galK rpsL endA1 nupG StrR* (39)] and MG1655 [Genotype: F-lambda-*ilvG*-*rfb*-50 *rph*-1 (40)]. Two of the three strains contained further adjustments to their genome. MDS42 has undergone CRISPR-mediated deletion of the *recA* gene to discourage unexpected homologous recombination. TOP10 has had its *lacZ* gene repaired using homologous recombination to allow for screening based on lactose fermentation on MacConkey agar with lactose. Each of these strains was grown overnight (12–18 h) in Luria Broth-Lennox medium with selective antibiotics (if applicable, for plasmid selection) at 32°C. For plating, overnight cultures were plated as dilutions on either Luria Broth-Lennox agar plates (if lactose fermentation is not being screened) or MacConkey agar with lactose plates (if lactose fermentation is being screened). Plates were incubated for 1–6 days (depending on strain fitness) at 32°C.

### Oligonucleotides used

A complete list of oligonucleotides used in this project is provided in Supplementary Table S1. Kanamycin resistance cassette amplification primers and MASC primers were designed and described in a previous work (1). Primers were designed manually or using Geneious Prime 2022.0.2 Primer Design software to a T_m_ of 60–61°C. MAGE oligonucleotides of 90 bp were designed to have a folding free energy between 0 and –15 kcal/mol, two phosphorothioated bonds on the 5′ ends and have the desired mutation as centered as possible within the oligonucleotide, as per the literature (28). The *lacZ*-off oligonucleotides were designed to create a T35G mismatch in the *lacZ* gene, generating a V11* nonsense mutation featured in other recombineering projects (24,26,27).

### Plasmids used

Synthesis and assembly of the 50 kb recoded segments from 2–4 kb Genebytes was described in a progress report on *rE.coli*-57 (1). These recoded segments were assembled on the pYES1L-URA BAC (Addgene #84301) to allow both for growth in *Saccharomyces cerevisiae* and low copy number (1–2 copies per cell) maintenance in *E. coli*. The pYES1L-URA BAC is spectinomycin selectable in *E. coli*. Kanamycin deletions to test complementation of recoded segments contained in a BAC were performed using pKD78, a chloramphenicol-selectable recombineering plasmid containing the three lambda-Red genes *exo*, *beta* and *gam* activated through arabinose induction (1,41).

Two recombineering plasmids were used during this project for ssDNA oligonucleotide recombineering. The chloramphenicol-selectable pORTMAGE-4 recombineering plasmid (Addgene #72679) contains the three lambda-Red genes and the dominant negative *mutL*E32K gene to inactivate the mismatch repair system transiently expressed using temperature induction (25). Finally, the gentamicin-selectable pORTMAGE-503B recombineering plasmid (derived from pORTMAGE-Ec1, Addgene #138474) contains the CspRecT SSAP and the dominant negative *mutL*E32K gene transiently expressed using *m*-toluic acid induction (26).

### Transformation protocols

**Figure 1.**
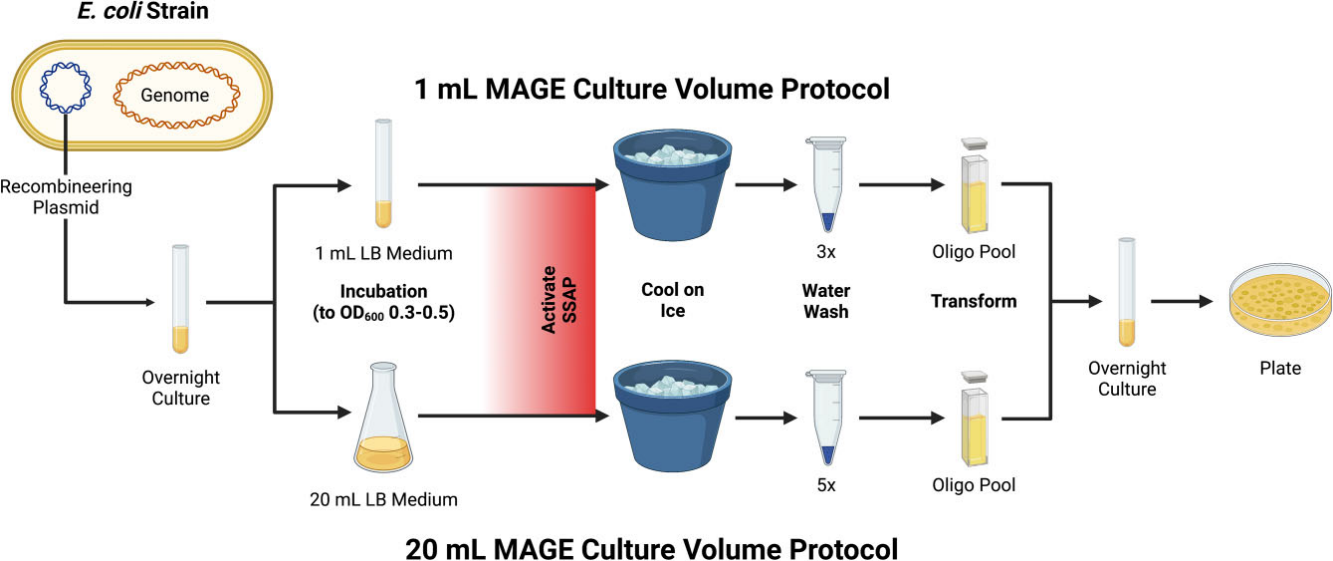
Overview of the recombineering protocol. Oligonucleotides of 90 bp were recombineered into the strain of interest, an *E. coli* strain with a recombineering plasmid (either pORTMAGE-4 or pORTMAGE-503B) and, for certain applications, a 50 kb recoded segment on the pYES1L-URA BAC. Here, 1 ml (three washes in water) or 20 ml (five washes in water) MAGE protocols were used. Overnight recovery cultures were plated on Luria Broth-Lennox agar with antibiotic selection according to the pORTMAGE plasmid present, and repaired colonies were identified and quality checked with Sanger sequencing and next-generation sequencing (NGS), respectively. The figure was created with BioRender.com.

Transformation of electrocompetent bacteria was performed using protocols previously described in the literature (Figure 1). For transformation of recombineering plasmids, kanamycin resistance cassettes and, for early recombineering experiments, 90 bp ssDNA oligonucleotides, a 1 ml transformation protocol described in Gallagher *et al.* was used (28). A 3 ml aliquot of Luria Broth-Lennox medium with selective antibiotics (if applicable, for the recombineering plasmid) was inoculated with the transformant strain and grown overnight (12–16 h) at 32°C. The next day, a 1:100 dilution of this overnight culture was prepared in 3 ml of Luria Broth-Lennox medium with selective antibiotics (if applicable) and grown at 32°C to OD_600_ 0.3–0.5, as determined by spectrophotometry. If applicable, SSAP activation then proceeded, and the culture was chilled on ice for 20 min. Then 1 ml of the culture was washed three times with chilled ultra-pure water, and, after the third wash, the cell pellet was resuspended in 80 μl of chilled ultra-pure water, and 2–4 μl of the DNA to be transformed was added. A 42 μl aliquot of the DNA:cell mixture was then added to a chilled 0.1 cm electrocuvette, and electroporated at 1.80 kV, 200 Ω, 25.0 μF. Cells were then recovered overnight in 1 ml of Luria Broth-Lennox medium and plated on Luria Broth-Lennox agar with selective antibiotics for 1–2 days at 32°C.

For transformation of 90 bp ssDNA oligonucleotides, a 20 ml transformation protocol described in Nyerges *et al.* was also used, with the increased cell pellet size found to be more researcher friendly (25). A 3 ml aliquot of Luria Broth-Lennox medium with selective antibiotics for the recombineering plasmid was inoculated with the transformant strain and grown overnight at 32°C. The next day, a 1:100 dilution of this overnight culture was prepared in 25 ml of Luria Broth-Lennox medium with selective antibiotics and grown at 32°C to OD_600_ 0.3–0.5, as determined by spectrophotometry. SSAP activation then proceeded, and the culture was chilled on ice for 20 min. Then 20 ml of the culture was pelleted and resuspended in 1 ml of chilled ultra-pure water. This pellet was then washed five times with chilled ultra-pure water, and, after the fifth wash, the cell pellet was resuspended in 80 μl of chilled ultra-pure water, and 2–4 μl of the DNA to be transformed was added. A 42 μl aliquot of the DNA:cell mixture was then added to a chilled 0.1 cm electrocuvette, and electroporated at 1.80 kV, 200 Ω, 25.0 μF. Cells were then recovered overnight in 1 ml of Luria Broth-Lennox medium and plated on Luria Broth-Lennox agar with selective antibiotics for 1–2 days at 32°C.

A variation to resuspension of the final cell pellet was introduced to test for the impact of genomic background, oligonucleotide direction, SSAP selected and cell count transformed. For testing the impact of genomic background, oligonucleotide direction and SSAP selected, the 20 ml transformation protocol was used, and, following wash steps, the final cell pellet was instead resuspended in 500 μl of water. An unpaired two-sample *t*-test was performed to compare conditions in GraphPad Prism, with *P*-value correction by the Holm–Šídák method and the *P*-value threshold set to <0.05. For testing the impact of cell count transformed on recombineering efficiency, this 500 μl water resuspension volume was varied over a range of 100 μl to 1.3 ml. For both experiments, 240 μl of the cell:water resuspension was added to one or more new microcentrifuge tubes, and 12 μl of 500 μM oligonucleotide stock was added. Each 252 μl of DNA:cell mixture was broken into five reactions of 42 μl into separate 0.1 cm electrocuvettes. Cell count transformed was calculated based on cell density per ml, outgrowth volume (20 ml), resuspension volume (intentionally varied over the range described) and volume of DNA:cell mixture added to each cuvette. Although cell division continues during activation, cell density was measured prior to SSAP activation, because of the importance of cell density measurements to determining when to activate the SSAP. Following recovery and plating, the total red and white colony count of each of these five reactions was combined as one replicate to increase the cells counted per reaction and to control for variability introduced by technical error on recombineering efficiency. Data were assessed using a non-linear fit to a quadratic function.

For colony sequencing, primers were manually generated to amplify the locus targeted for repair, and primers were designed to be allele specific to the recoded segment so that the corresponding genomic segment would not be sequenced. Polymerase chain reaction (PCR) was performed using 2GMP on the 30–192 post-recombineering colonies individually (average of 89 edited cells per sequencing run) and a wild-type control (here TOP10 or MDS42 for segment 12) to amplify the target locus and confirm allele specificity, respectively. Target DNA was sent to Genewiz (now Azenta Life Sciences) for Sanger sequencing of unpurified PCRs.

For lactose fermentation screening, recovery cultures were plated on MacConkey agar containing lactose. Here, successful recombinants were unable to ferment lactose and resulted in white colonies (compared with lactose fermenters producing red colonies). Following recombineering and screening for white colonies on MacConkey agar medium containing lactose, recombineering efficiencies were calculated by dividing the number of white colonies by the total number of colonies and multiplying by 100.

### Recoded segment complementation and analysis

Complementation of corresponding wild-type deletion by a recoded segment contained in a BAC using a single kanamycin resistance cassette was tested using the methods and primers described in a previous work (1). A kanamycin resistance cassette with ∼50 bp homology arms to the loci immediately flanking the corresponding wild-type region was PCR amplified and gel purified. Then, an *E. coli* strain containing the recoded segment of interest on the pYES1L-URA BAC and the pKD78 recombineering plasmid was transformed with the purified kanamycin resistance cassette, recovered overnight and plated on Luria Broth-Lennox agar containing kanamycin.

Determination of the result was based on the presence of colonies and the result from PCR testing. If colonies were not present for the whole segment deletion, the segment was diagnosed as requiring further troubleshooting to complement wild-type deletion. If colonies were present, MASC-PCR was performed on colonies to check for presence of the corresponding wild-type and recoded loci, as described in Ostrov *et al.* (1). If both wild-type and recoded bands were fully present (eight bands indicating full presence of the corresponding locus), the segment was diagnosed as requiring further troubleshooting to complement wild-type deletion (1).

### NGS and data analysis

The recoded segment BACs were purified from an overnight 3–5 ml culture of the TOP10 *E. coli* strain containing the recoded segment on a pYES1L-URA BAC. BAC preps of the 10 segments described were sent to MiSeq for NGS to generate unpaired and paired 150 bp reads. Following read generation, read files were uploaded to Geneious Prime 2022.0.2 (http://www.geneious.com/). Reads were then processed using the Geneious ‘Trim and Filter’ workflow, set to ‘Annotate new trimmed regions’ with ‘Error Probability Limit’ set to 0.05, and ‘Trim 5′ End’ and ‘Trim 3′ End’ selected. Filtered and trimmed reads were then aligned to recoded segment files using the Bowtie2 Geneious plug-in, local alignment setting (42).

### Computational analysis of protein structure predictions

Protein structure predictions were obtained using the DeepMind AlphaFold CoLab, running AlphaFold2.1.0. Protein amino acid sequences were input, and the notebook was set to ‘is_prokaryote’ and ‘run_relax’ settings. Runs were performed using a Google CoLab Pro + account, with ‘High Ram Run’ and ‘Run in Background’ selected. Two protein alignments were generated for each wild-type and mutated protein pair to generate the root mean square deviation (RMSD) for each (with higher RMSD values indicating more dissimilarity between the protein structures) and the template modeling score (TM-score) for RCSB (values ranging from 0 to 1, with 1 indicating that the protein structures are identical). First, wild-type and mutated protein versions were aligned using the RCSB Pairwise Structure Alignment tool, employing the jFATCAT (rigid) algorithm. Next, wild-type and mutated protein versions were aligned using the PyMOL align command, running for five cycles to remove outlier atoms.

### Computational analysis of protein sequence conservation predictions

Protein sequence conservation predictions were obtained using the DDGun web interface (43). Protein amino acid sequences were input, as well as mutation amino acid position, original amino acid identity and mutated amino acid identity. DDGun takes computed differences between the original and mutated amino acid at the position (the BLOSUM62 evolutionary conservation score, and the change in interaction energy and hydrophobicity between the two residues), and reports the change in stability (ΔΔ*G*, kcal/mol) between the two amino acids (43). Further, based on the multiple sequence alignment generated by the tool, the difference in frequency of finding the two amino acids at the same position is also reported (43). To compare the frequency of the wild-type amino acid versus the mutated amino acid, the ratio was taken of the two reported values.

## RESULTS

### Testing protocol parameters impacting recombineering efficiency

#### Testing the impact of E. coli genome, SSAP selected and oligonucleotide direction on recombineering efficiency

During whole-genome engineering work, the need to test changes to the genome can occur at any locus, and researchers must be prepared to edit recombineering-recalcitrant loci by constantly testing improvements to the strain repair process. Due to researcher-specific discrepancies between maximum recombineering efficiencies, as well as the use of multiple *E. coli* strains and SSAP plasmids during the *rE.coli*-57 genome engineering process, it was important to establish recombineering efficiencies of different tools used to determine which resulted in improved performance. To determine the impact of strain characteristics on recombineering efficiency and to select characteristics resulting in the highest recombineering efficiency, different combinations of *E. coli* genomes, recombineering plasmids and oligonucleotide directions were tested. Three common *E. coli* genomes employed in genetic and genomic engineering work were used as the basis of strain construction: MDS42, TOP10 and MG1655 *E. coli* (38). For each of the three *E. coli* strains, two recombineering plasmids from the pORTMAGE system were incorporated via transformation: pORTMAGE-4 and pORTMAGE-503B. These plasmids were selected as each carried a different SSAP tested in the literature. While pORTMAGE-4 expresses the lambda-Red Beta SSAP, pORTMAGE-503B expresses the CspRecT SSAP (25,26). The six strains constructed underwent a single cycle of recombineering to introduce a premature stop codon into the genomic *lacZ* gene, using either a forward or a reverse direction version of the oligonucleotide to confirm the impact of oligonucleotide direction. Introduction of the premature stop codon via recombineering was screened for on MacConkey agar plates containing lactose.

Based on the scientific literature, we generated three hypotheses. First, the MG1655 *E. coli* strain will produce a higher recombineering efficiency than the TOP10 or MDS42 *E. coli* strains, as MG1655 has the highest fitness of the three strains, has the fewest deleted genes (more pathways exist to bypass a deleterious mutation) and is commonly used for checking recombineering efficiency in *E. coli* (25,26,41,44). Second, strains using the CspRecT SSAP will produce higher recombineering efficiencies than those using the Beta SSAP, as CspRecT is a high recombineering efficiency SSAP identified using a SSAP serial enrichment protocol (25,26). Third, the reverse direction oligonucleotide will produce a higher recombineering efficiency than the forward direction oligonucleotide, as *lacZ* is on the first replichore of the *E. coli* genome on the negative strand and lagging strand-targeting oligonucleotides (28).

**Figure 2.**
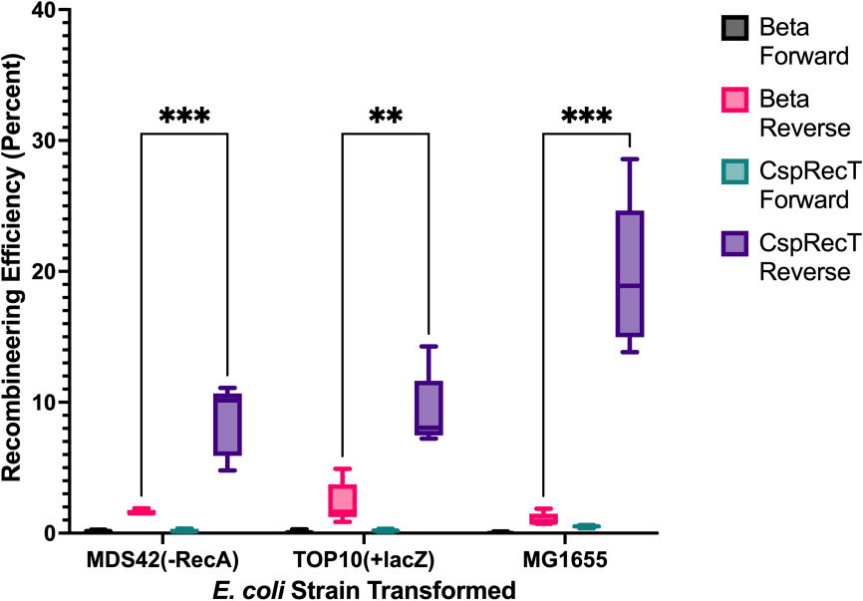
Impact of *E. coli* genome, SSAP selected and oligonucleotide direction on recombineering efficiency. One cycle of recombineering was performed to introduce a premature stop codon into the *lacZ* gene, recovered overnight in Luria Broth-Lennox medium and plated on MacConkey agar with lactose. Recombineering was performed targeting the genomic copy of *lacZ* in three *E. coli* genomes: MDS42(-*recA*), TOP10 (with *lacZ* repaired) and MG1655. An identical oligonucleotide was developed in the forward and reverse directions. While the pORTMAGE-4 plasmid was used to supply the Beta SSAP, the pORTMAGE-503B plasmid was used to supply the CspRecT SSAP. For each strain, pORTMAGE plasmid and oligonucleotide direction, five replicates were performed. Whiskers are the minimum and maximum data points on the chart for each condition. **P< 0.01 and ***P< 0.001. The figure was created with GraphPad Prism.

Through characterization of the impact of *E. coli* genomic background, SSAP selected and oligonucleotide direction, we were able to systematically confirm literature predictions for these factors. For all genomic background and SSAP combinations tested, the reverse direction oligonucleotide resulted in significantly higher recombineering efficiency than the forward direction oligonucleotide for genomic *lacZ* (Figure 2, *P* < 0.02). Beyond the oligonucleotide direction, the CspRecT SSAP resulted in higher recombineering efficiency for all *E. coli* strains when compared with their counterpart containing the Beta SSAP (*P* < 0.002). Overall, we saw that the MG1655 *E. coli* strain containing the CspRecT SSAP on the pORTMAGE-503B plasmid resulted in the highest recombineering efficiency when the oligonucleotide was properly designed as a reverse direction oligonucleotide to target the lagging strand for DNA replication. Interestingly, a previous study indicated that MDS42 outperformed TOP10 and MG1655 for plasmid uptake during transformation, indicating that the reduced recombineering efficiency seen here for MDS42 and TOP10 is specific to DNA incorporation into the genome (38).

#### Testing the impact of cell density at transformation on recombineering efficiency

**Figure 3.**
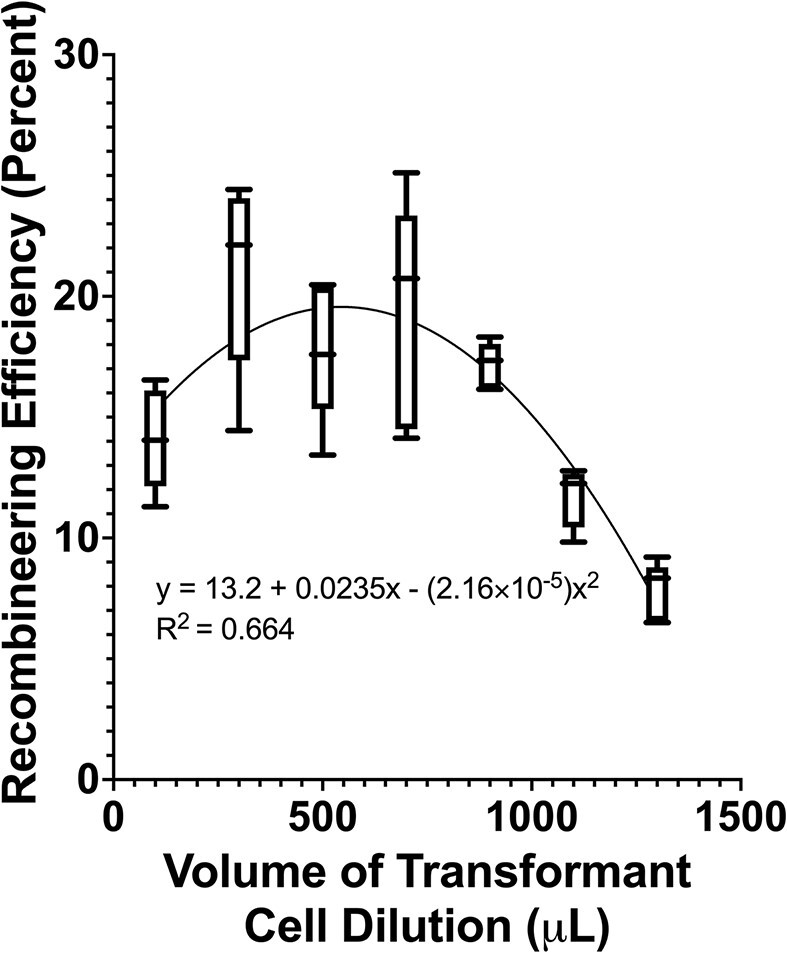
Impact of cell density at transformation on recombineering efficiency. MG1655 *E. coli* with pORTMAGE-503B underwent one cycle of recombineering using the reverse direction oligonucleotide introducing the premature stop codon into the genomic *lacZ* gene. Different cell dilutions in water were used to determine the optimal cell dilution at transformation. Five replicates of each dilution volume (except for the 1100 μl dilution, which had four replicates) were performed in this experiment, with seven dilution volumes tested. Whiskers are the minimum and maximum data points on the chart for each condition. The figure was created with GraphPad Prism.

As the MG1655 *E. coli* strain, CspRecT SSAP and reverse direction oligonucleotide combination resulted in the highest recombineering efficiency when targeting the genomic *lacZ* gene, these parameters were then used to test the impact of cell density at transformation on recombineering efficiency (Figure 1). By resuspending cells outgrown in similar conditions in volumes of water ranging from 100 to 1300 μl, we found that between 300 and 700 μl (or ∼3.20 × 10^8^ to 8.32 × 10^8^ cells per cuvette) results in the highest recombineering efficiency (Figure 3).

Cell density at transformation was chosen as a parameter to test, because increasing the amount of water in which the final cell pellet is diluted increases the number of possible transformations from a single washed culture, assuming all other protocol conditions remain constant. With the final cell pellet diluted in 300–700 μl of water from a 20 ml outgrowth culture, this result demonstrates that up to 17 parallelized recombineering reactions can be performed simultaneously to obtain a high recombineering efficiency with a minimal increase in effort compared with that needed for one recombineering reaction, as only the steps following the MAGE cell pellet wash expand to accommodate multiple transformations. Furthermore, while increasing the volume of water in which the final cell pellet is diluted beyond 700 μl does lower the recombineering efficiency, further dilution increases the number of parallelized recombineering reactions possible for a single 20 ml culture. For dilution in 900 μl of water, recombineering efficiency is at an average of 17.2%, with a 900 μl dilution allowing for 22 parallelized recombineering reactions. Further dilution may even be possible for beneficial mutations, such as those resulting in overcoming antibiotic selection for growth on media plates.

### BAC recombineering to repair mutations in episomal *de novo* synthesized DNA segments

#### Requirement for BAC recombineering in rE.coli-57 strain construction

Rigorous optimization of the recombineering protocol has many possible applications, with an important one being the construction of synthetic genomes. Here, we apply recombineering to editing BACs containing MDS42 *E. coli* genome segments towards the development of a recoded *E. coli* strain (*rE.coli*-57). To determine whether recoded segments were able to complement wild-type deletion, recombineering-mediated deletion with a single antibiotic resistance cassette was performed to remove the corresponding wild-type genes in the TOP10 host genome (1). For recoded segments unable to complement wild-type deletion, NGS of the synthesized segment contained in a BAC was used to identify <30 bp non-recoding mutations present in recoded essential genes (45). In this manner, 10 recoded segments were identified as candidates for non-recoding mutation repair with 22 non-recoding mutations <30 bp. While these mutations included five small deletions (<30 bp), the remaining 17 mutations were SNPs in protein-coding sequences, resulting in one nonsense mutation, three silent mutations and 13 missense mutations (the full list of mutations identified is given in Supplementary Table S2). As the recoded segment was contained on a BAC for transfer from *S. cerevisiae* into *E. coli*, BAC recombineering was identified as the strategy to use for non-recoding mutation repair (1). This BAC recombineering would be performed on recoded segment copies contained on BACs in a TOP10 *E. coli* strain, with a minimum of two target copies present per strain as the wild-type copy of the segment could not be deleted prior to recombineering, thus necessitating higher efficiency recombineering despite the need to obtain only one strain with the recoded segment successfully repaired.

#### Non-recoding mutation repair strategy

BAC recombineering was used to repair SNPs and small deletions ranging from 1 to 30 bp in 10 synthesized, recoded segments using the pORTMAGE recombineering plasmid system. For nine segments, non-recoding mutations present in recoded essential genes were targeted for repair on a segment copy contained on a BAC in a TOP10 *E. coli* host, as TOP10 is an *E. coli* strain suited to maintaining clonal DNA. For one segment (segment 12), repair was instead performed on the same BAC in an MDS42(-*recA*) strain, as the TOP10 genomic locus containing the segment genes overlapped with a large repeat region not present in the MDS42 strain.

#### Single oligonucleotide BAC recombineering to repair a dnaG missense mutation

**Figure 4.**
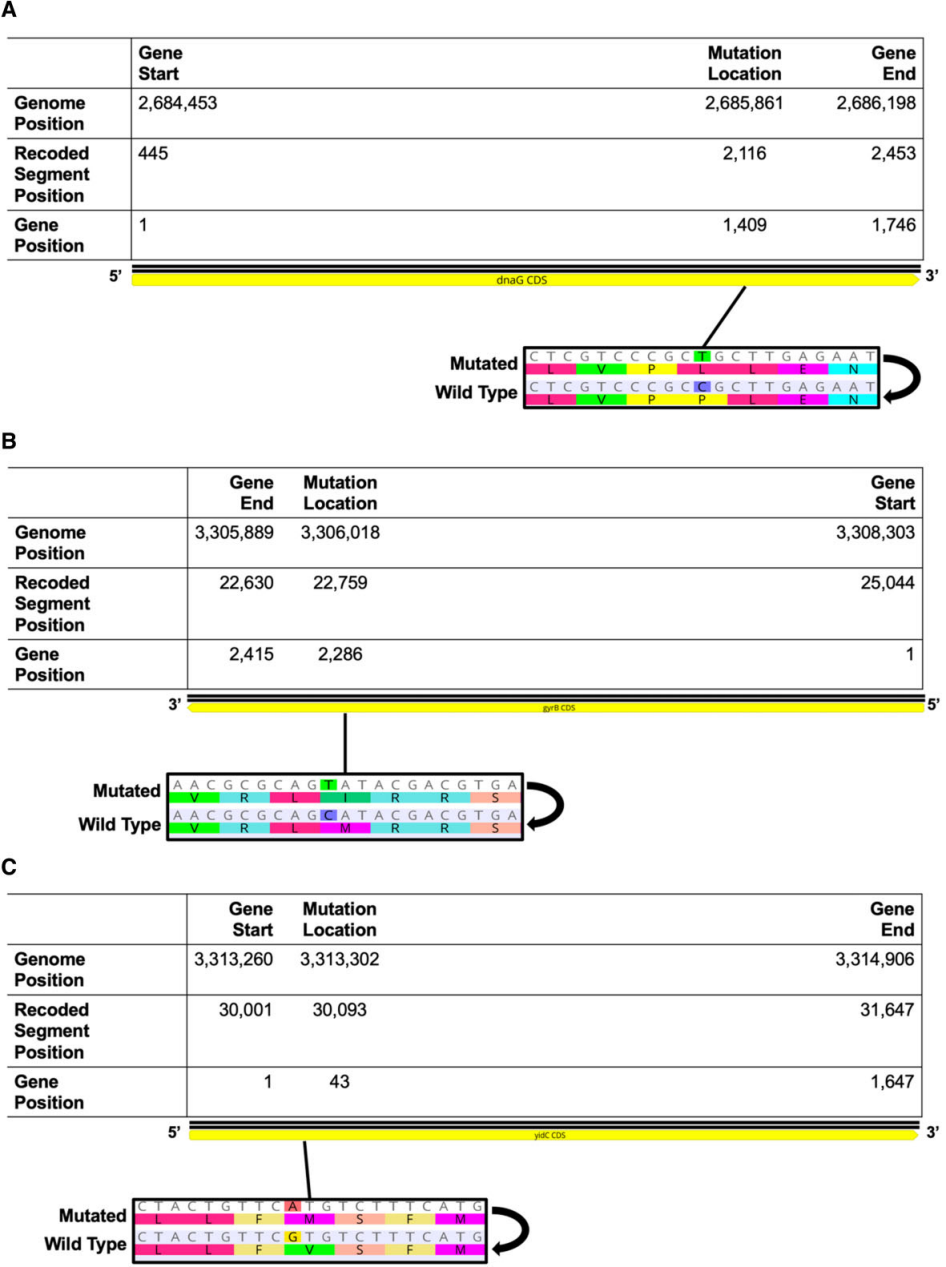
NGS identifies SNPs in essential genes in recoded segments 59 and 72. (**A**) The recoded *dnaG* gene in segment 59 contains a C to T SNP, resulting in a P470L missense mutation in the DnaG protein product. (**B**) The recoded *gyrB* gene in segment 72 contains a C to T SNP, resulting in a M762I missense mutation in the GyrB protein product. (**C**) The recoded *yidC* gene in segment 72 contains a G to A SNP, resulting in a V15M missense mutation in the YidC protein product. Genome and segment positions are given based on the published *rE.coli-*57 genome (1). The figure was created with Geneious Prime 2022.0.2 and Biorender.com.

One of the recoded segments repaired was recoded segment 59. While segment 59 contains four essential genes (*rpsU*, *dnaG*, *rpoD* and *higA*), one essential gene [*dnaG* (encoding the DNA primase protein)] was found to contain a non-recoding mutation based on NGS (Figure 4A). The P470L missense mutation in the DnaG primase protein is located in the C-terminal domain of the protein, specifically within the hydrophobic pocket that interacts with the C-terminal tail of the SSB protein (46).

The mutation was repaired on the recoded segment 59 contained in a BAC using one cycle of MAGE in a TOP10 strain with pORTMAGE-4 as the recombineering plasmid, with a recombineering efficiency of 3.2%. Following recombineering-mediated repair, Sanger sequencing was used to obtain a copy of the recoded segment with the missense mutation repaired in *dnaG*, and NGS was used to confirm no additional missense mutations were present in recoded essential genes following repair. Complementation by the whole recoded segment was then tested, and it was found that the repaired segment 59 could now complement wild-type deletion.

#### Multiple oligonucleotide BAC recombineering to repair gyrB and yidC missense mutations

While the repair of *dnaG* in recoded segment 59 served as an example where only one missense mutation was present in a recoded essential gene in a segment, the average number of mutations repaired with BAC recombineering per 44 402–47 179 bp segment was 2.2 mutations. Recoded segment 72 was a synthesized DNA segment for which multiple non-recoding mutations were repaired simultaneously, here two repairs. While segment 72 contains six essential genes (*gyrB*, *dnaN*, *dnaA*, *rpmH*, *rnpA* and *yidC*), only two were found to contain non-recoding mutations: *gyrB* (encoding the DNA gyrase subunit B protein) and *yidC* (encoding a membrane protein insertase) (Figure 4B, C). The M762I missense mutation in GyrB was in the C-terminus of the protein, outside of catalytic domains. Meanwhile, the V15M missense mutation in YidC was located in the N-terminus of the protein, in the first transmembrane domain signal-anchor sequence (47).

The *gyrB* and *yidC* missense mutations were repaired simultaneously with an oligonucleotide pool on the recoded segment 72 contained in a BAC. Four cycles of MAGE were performed in a TOP10 strain with pORTMAGE-503B as the recombineering plasmid, with recombineering efficiencies of 23.4% for *gyrB* and 28.1% for *yidC*. Repair of the mutation was confirmed as described for segment 59. However, although recoded segment 72 could now complement wild-type deletion, it did so with decreased fitness, indicating that other avenues for segment troubleshooting remain to be explored. We hypothesize that troubleshooting of the recoding scheme for the segment will lead to further fitness improvements for segment 72 complementation. By performing BAC recombineering on recoded segment 72 with the wild-type copy of the segment deleted to test candidates for improvement, later research can take advantage of the presence of only the recoded segment for improvements.

### Assessing use of protein structure prediction to prioritize non-recoding mutation repair with test segments

Following completion of the repair of 22 non-recoding mutations in the essential genes of 10 recoded segments, we were interested in whether currently available computational programs could allow future efforts for large-scale genome engineering to prioritize repair of unexpected mutations, particularly those resulting in missense mutations. While working on this project, the DeepMind team released AlphaFold, allowing for robust protein structure predictions from amino acid sequences (48,49). Although this enormous undertaking represents a significant leap forward in computational biology, recent work has indicated the need for cautious optimism regarding the use of AlphaFold to predict the impact of individual mutations on a protein, encouraging researchers to bear in mind that protein function and protein structure are not equivalent (50,51).

Here, we look at predictions made regarding protein structure on the two example segments described above. In the first example, repair of a single missense mutation in the *dnaG* of segment 59 improved complementation fitness. However, while repairing two missense mutations in recoded segment 72 simultaneously improved fitness, the impact of these individual mutations on recoded segment complementation fitness is unknown. Here, we tested the use of AlphaFold to predict whether it was the repair of one or both mutations that improved complementation fitness. We further tested two deleterious missense mutations previously identified in the literature as controls: mutation of the autophosphorylated serine in the serine/threonine kinase HipA (S150A), and a dominant lethal mutation of the –35 recognition site of sigma70 protein RpoD (E585Q) (52,53).

**Figure 5.**
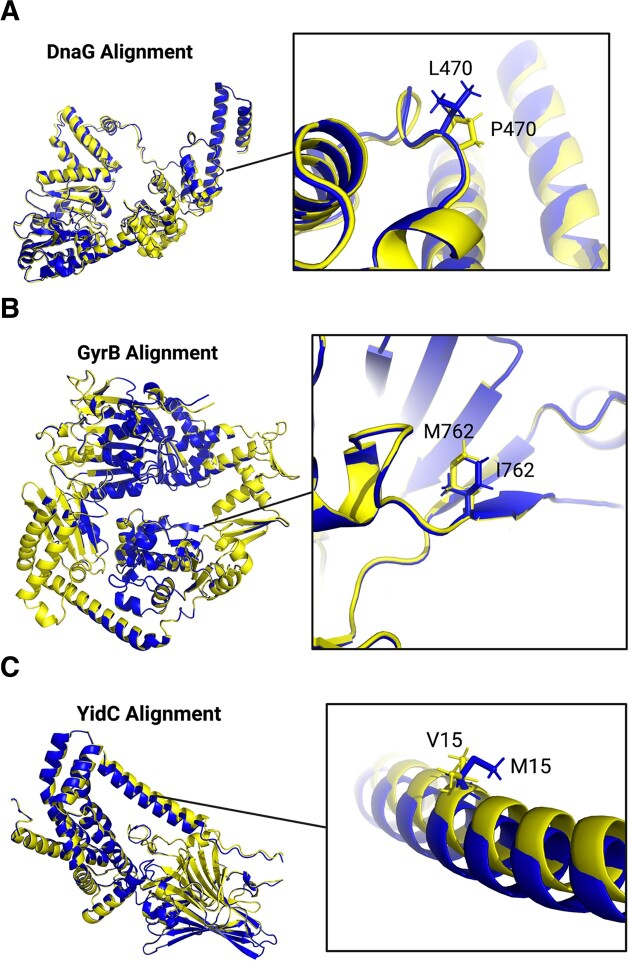
Protein structure prediction alignments for wild-type and mutated DnaG, GyrB and YidC. Protein structure prediction PDB files obtained from the AlphaFold2 CoLab Notebook were aligned using PyMOL. Wild-type structures are shown in yellow, and mutated structures are shown in blue. Wild-type and mutated residues are shown as insets, with zoom set to 12 Å. (**A**) DnaG wild-type (P470) and mutated (L470) protein structure alignment. (**B**) GyrB wild-type (M762) and mutated (I762) protein structure alignment. (**C**) YidC wild-type (V15) and mutated (M15) protein structure alignment. The figure was created with PyMOL and Biorender.com.

Wild-type and mutated protein structure predictions were aligned using RCSB Pairwise Structure Alignment (using a jFATCAT-rigid alignment) and PyMOL, and protein structure similarity was assessed using RMSD values (Figure 5). Importantly, while RCSB Pairwise Structure Alignment reports RMSD without removing outlier atoms, PyMOL align reports RMSD both before and after a set number of cycles filtering for outlier atoms. Here, we report the RCSB Pairwise Structure Alignment RMSD, as well as both the PyMOL RMSD values before outlier filtering and after five cycles of outlier filtering (Table 1). As the RMSD values are impacted by sequence length, TM-score was also used to determine whether sequence length impacted our ability to use protein structure predictions to test the impact of single residue changes on protein structure (54). For four of the five proteins (DnaG, YidC, GyrB and HipA), all RMSD values generated were very low, indicating that the protein structures compared in the alignments were very similar. Similarly, TM-scores for these four proteins were very close to or equal to 1, confirming that, even accounting for sequence length, the alignments are very similar. This was expected due to the high degree of protein sequence homology between mutated and wild-type protein sequences.

**Table 1.** RMSD and TM-score for DnaG, YidC and GyrB wild-type and mutated predicted protein structure alignments, with HipA and RpoD known deleterious missense mutation controls

Protein name	Protein length (amino acids)	RCSB protein alignment RMSD (Å)	PyMOL protein alignment RMSD (Å)		TM-score
			0 Cycles	5 Cycles	
DnaG	581	0.75	0.75	0.63	0.99
GyrB	804	0.35	0.35	0.23	1
YidC	548	0.66	0.66	0.24	0.99
HipA	440	0.14	0.82	0.089	1
RpoD	613	4.6	11	2.7	0.73

For one of the proteins (RpoD), the missense mutation resulted in a more observable change in the protein alignments, although five cycles of PyMOL outlier filtering do decrease the RMSD to below the cut-off for highly similar proteins (Table 1). We hypothesize that this is due to the difference in hydrophobicity resulting from the RpoD E585Q mutation, with hydrophobicity being an important factor in protein folding (for detailed data generated by DDGun, see Supplementary Table S3) (43,55). While it is promising to see that one of the mutant proteins is able to be identified from its wild-type counterpart using the AlphaFold CoLab Notebook, protein structure was not viable to identify the four other protein mutants selected for study. Further, towards the goal of prioritizing which mutation to repair for recoded segment 72, this tool did not allow us to distinguish between the YidC and GyrB mutations and considered the wild-type and mutant versions of these proteins to be too similar to distinguish.

### Assessing use of protein sequence conservation prediction to prioritize non-recoding mutation repair with test segments

As protein structure should not be overgeneralized to protein function, we tested whether we could distinguish between the mutant and wild-type sequences with computational tools more predictive of protein function. While tools exist to computationally predict protein function (56–59), these tools have command-line interfaces and can be intimidating for users to start working with. As protein stability and sequence conservation have both been implicated in identification of variants impacting protein function, we sought to use a tool that addressed both factors to distinguish between variants: DDGun (43) Wild-type protein sequences were run through the sequence-only section of the DDGun web server with the amino acid mutation of interest to generate protein stability and conservation data (Table 2). While we observed that protein stability changes between the wild-type and mutant protein sequence were variable (and indeed some mutations were predicted to improve protein stability over the wild type), the frequency of observing the wild-type amino acid versus the mutant amino acid in the multiple sequence alignment was more informative. For both the literature-generated mutations, the ratio of the frequency of the wild type to the mutated amino acid was >1, indicating that the wild type amino acid was more conserved than the mutant. Further, sequence conservation allows us to distinguish between the YidC and GyrB mutations in recoded segment 72, with the YidC wild type being much more conserved compared with its mutant than GyrB. This represents a possible mutation for follow-up away from the context of strain engineering work.

**Table 2.** DDGun predictions of protein stability and amino acid conservation for DnaG, YidC and GyrB wild-type and mutated predicted protein sequences, with HipA and RpoD known deleterious missense mutation controls

Protein name	Amino acid mutation	ΔΔ*G* (kcal/mol)	Frequency of wild-type amino acid	Frequency of mutant amino acid	Ratio of wild-type to mutated amino acid frequency
DnaG	P470L	0.4	1.9	2.4	0.79
GyrB	M762I	0.1	6	3.2	1.88
YidC	V15M	-0.3	9.2	0.6	15.33
HipA	S150A	-0.4	57.5	4.5	12.78
RpoD	E585Q	0	36.9	4.6	8.02

Interestingly, the DnaG P470L mutation (the only intentional repair made to recoded segment 59 on the BAC) demonstrated the opposite effect, with the mutant amino acid being more conserved than the wild type, and the protein stability predicted to improve with the mutation (Table 2). We hypothesize that this could be due to three possible reasons. First, DnaG P470L could have been the causative mutation, and the multiple sequence alignment for DnaG may not be robust, meaning there is more variability at the locus when stability and conservation predictions are being generated. Second, the DnaG missense mutation may not have been the cause of poor fitness in the recoded segment 59 strain, and another unknown cause may have contributed for which a strain with a suppressor for the cause was identified during segment 59 repair (such as a strain with a deleterious SNP in another part of the genome obtaining a suppressor mutation elsewhere). Third, another factor could be more important to predict which mutations should be prioritized for repair, representing another avenue for future work.

## DISCUSSION

Here, we tested four parameters of the recombineering protocol to increase recombineering efficiency for ssDNA oligonucleotide incorporation into the *E. coli* genome, with the goal of demonstrating a robust recombineering protocol for repairing 22 non-recoding mutations in ten 44 402–47 179 bp recoded segments contained on BACs in the r*E.coli*-57 strain engineering project. As anticipated when starting this project, repair of non-recoding mutations improved fitness for some, but not all, recoded segments described, thus indicating that other problems may contribute to reduced segment fitness (such as the recoding scheme itself). For this work, we repaired non-recoding mutations in essential genes, but acknowledge that different methods of identifying essential genes result in some variation in the list of genes defined as essential, and gene essentiality cannot be categorized in a binary manner (60–62). Additionally, for many applications of complementation testing for synthetic DNA segments against wild-type counterparts, complementation by the synthetic DNA segment may not entirely eliminate fitness, but rather reduce fitness compared with the wild type. While the segments of interest in this study were those for which complementation by the recoded segment caused fitness loss, it would be possible to use a similar strategy to that described herein to repair segments for which complementation reduced, but did not eliminate, fitness. Further, as this work was done in the context of constructing a fully recoded *E. coli* strain, the non-recoding mutations identified and repaired were not the only changes made to the MDS42 genome. Previous efforts have identified possible means by which recoding may require further troubleshooting, including changes to mRNA folding and ribosomal binding sites (4,10,63). While identification of individual recoded codons impacting fitness is a non-trivial task, the BAC recombineering strategy described in this work could be used to repair both recoding and non-recoding mutations contributing to decreased fitness.

Quantification of fitness using a plate reader growth assay would be optimal to determine the extent to which repair efforts improve fitness of a strain forced to rely on the synthetic segment compared with its wild-type counterpart. However, the plate reader method was not used in this study due to the context of the work. The segment repair work occurred in a larger pipeline, the goal of which was to ready recoded genomic segments for incorporation into the *rE.coli*-57 genome. A necessary trade-off in strain engineering work is made between testing the biological ramifications of individual mutations versus timely delivery of a strain. However, as strain engineering work leads to identification and repair of causes of decreased complementation fitness, it is even more important to report such findings, allowing labs more focused on testing individual mutations to study variants that would otherwise require mutational screening of the locus of interest for their discovery. Identification and repair of further causes of decreased complementation fitness is an exciting challenge, with the possibility of uncovering novel biological rules.

Computational tools are a powerful means to generate hypotheses that can be tested at the bench. Here, we sought to generate hypotheses regarding why identified missense mutations found to decrease complementation fitness are deleterious, looking to determine whether protein structure and function were impacted by the missense mutations using AlphaFold and DDGun, respectively. While this tool was applied to a couple of examples in this work, AlphaFold's utility should be investigated for larger datasets with known links between phenotype and observed missense mutations (importantly, one group has already tested this with green fluorescent protein variants linked to fluorescence data) (51). In this work, we used a graphic user interface (GUI) version of AlphaFold on Google CoLab and the web server version of DDGun. Use of GUI tools allows anyone to pick up the tool, regardless of their computational background. In this manner, we sought to use available computational tools in such a way as to make them most available to anyone seeking to use them for their own applications to encourage people to enter the strain engineering field, regardless of their field of training. While GUI tools are more generally usable, they often allow for fewer simultaneous tests and may not provide the user with the same depth of data output as the command-line version. Therefore, when a larger number of tests or additional data outputs are required, users should familiarize themselves with the command-line versions. Further, limiting ourselves to GUI-based tools restricted what we could use for this study. There are many powerful computational tools such as UniRep and ESM-1V that do not have GUI versions at the time of this work to improve accessibility for early-stage strain engineers but could (and do) have exciting applications in strain engineering work (56–59). Further, as biology hypothesis generation increasingly relies on computational tools, it is important to recognize that, presently, much of our hypothesis testing must rely on benchwork (such as generating protein mutants and checking their structure through crystallography).

Improving protocols used in strain engineering can improve the speed and cost for whole-genome engineering work, critical as researchers look to expand the scope of projects being tackled (64,65). Such efforts also improve the accessibility of synthetic biology techniques for labs or research that may not be as well funded, critical especially to early-stage principal investigators, or research into domains of biology less immediately useful or outside of geographic biology hotspots, and therefore less likely to obtain substantial funding. As we move forward with large-scale genome engineering work, we must be cognizant that, at this time, the work we do is prohibitively expensive, in terms of cost, time and labor. Just as we, as synthetic biologists, hold biocontainment as a characteristic of responsible research, we must consider frequent communication about challenges and possible solutions being faced in this research important as well (66). In this manner, we can present problems faced to the general scientific community, where diverse perspectives can provide unique, creative solutions. Further, transparency provides researchers looking to perform large-scale genome engineering with an understanding of challenges faced during the process, which in turn allows them to better prepare and to set realistic timelines for strain completion.

## Supplementary Material

gkad692_Supplemental_Files

## Data Availability

Data are available on request. AlphaFold2.1.0 is available on Google CoLab for academic use (https://colab.research.google.com/github/deepmind/alphafold/blob/main/notebooks/AlphaFold.ipynb). BioRender is available for academic use (https://biorender.com). Bowtie2 is available on the Web for academic use (https://github.com/BenLangmead/bowtie2). DDGun is available on the Web for academic use (https://folding.biofold.org/ddgun/index.html). Geneious Prime 2022.0.2 is available for academic use (http://www.geneious.com/). GraphPad Prism 9.5.1 is available for academic use (https://www.graphpad.com). PyMOL Version 2.4.2 is available for academic use (https://PyMOL.org/2/). RCSB Pairwise Structure Alignment is available on the Web for academic use (https://www.rcsb.org/alignment).
