# Supplementary material for: Strategies to identify and edit improvements in synthetic genome segments episomally
Source: Nucleic Acids Res. 2023 Aug 24;51(18):10094–106. doi: 10.1093/nar/gkad692 (PMC10570025; doi:10.1093/nar/gkad692)
Supplement: gkad692_Supplemental_Files [file gkad692_supplemental_files.zip › Supplementary_Table_Legend.docx]

**Supplementary Table 1.** Oligonucleotides used in this study.

Oligonucleotides of 90 bp were transformed into *E. coli* as part of recombineering experiments. For *rE.*coli-57 recombineering oligonucleotides, names were formatted as “[segment number] – [gene targeted for repair].[base pair location on *rE.coli*-57 genome].fix – [direction of oligonucleotide]”. Oligonucleotides less than 90 bp were used as primers for PCR reactions to check for the presence of recombineering-mediated changes to the cell’s DNA content. For *rE.*coli-57 primers, names were formatted as “[segment number] – [gene targeted for repair].fix – [direction of primer]”. “*” in oligonucleotide sequences indicates a 5’ phosphorothioated bond.

**Supplementary Table 2.** Mutations present in essential genes in sample of ten recoded, BAC-contained segments.

Using NGS on BAC preps of ten recoded segments, non-recoding mutations were identified in all ten segments. As these segments were unable to complement deletion of their wild type counterpart, it was assumed that one or more mutations in essential genes may be the cause. All essential genes were manually checked for non-recoding mutations, and, if present, the type of mutation, the nucleotide impacted, and, for SNPs, the expected and actual nucleotide present were noted. If the mutation impacted the amino acid sequence, the type of change, and, for missense and nonsense mutations, the expected and actual amino acid were noted.

**Supplementary Table 3.** Full DDGun web server output comparing wild type and mutated sequences of five tested proteins.

The wild type and mutated sequences of three experimental (DnaG, GyrB, and YidC) and two known deleterious positive control (HipA and RpoD) proteins were run with the DDGun web server. DDGun output for each sequence comparison included the Kyte-Doolittle hydrophobicity score, the BLOSUM62 residue similarity score, profile (Skolnick) alignment score, protein stability (ΔΔG and Predictions[SEQ]), Kyte-Dolittle Hydropathy Index (dKD), and the wild type and mutant amino acid frequencies from the multiple sequence alignment generated by DDGun. For comparison of protein conservation between proteins, the ratio of wild type to mutated amino acid frequency was calculated separately.
